# Isolation and Characterization of New Bacteriophages against Staphylococcal Clinical Isolates from Diabetic Foot Ulcers

**DOI:** 10.3390/v15122287

**Published:** 2023-11-22

**Authors:** Lucile Plumet, Madjid Morsli, Nour Ahmad-Mansour, Fernando Clavijo-Coppens, Laurence Berry, Albert Sotto, Jean-Philippe Lavigne, Denis Costechareyre, Virginie Molle

**Affiliations:** 1 VBIC, INSERM U1047, University of Montpellier, 34095 Montpellier, France; lucile.plumet@umontpellier.fr (L.P.); nour.mansour@umontpellier.fr (N.A.-M.); 2 VBIC, INSERM U1047, Department of Microbiology and Hospital Hygiene, University of Montpellier, CHU Nîmes, 30908 Nîmes, France; mor_madjid@hotmail.com (M.M.); jean.philippe.lavigne@chu-nimes.fr (J.-P.L.); 3 Greenphage, Cap Alpha, 34830 Clapiers, Francedenis.costechareyre@greenphage.com (D.C.); 4 Laboratory of Pathogen and Host Immunity, CNRS UMR5294, University of Montpellier, 34095 Montpellier, France; laurence.berry@umontpellier.fr; 5 VBIC, INSERM U1047, Department of Infectious Diseases, University of de Montpellier, CHU Nîmes, 30908 Nîmes, France; albert.sotto@chu-nimes.fr

**Keywords:** bacteriophages, biofilm, diabetic foot ulcer, *Kayvirus*, *Staphylococcus* sp.

## Abstract

*Staphylococcus* sp. is the most common bacterial genus in infections related to diabetic foot ulcers (DFUs). The emergence of multidrug-resistant bacteria places a serious burden on public health systems. Phage therapy is an alternative treatment to antibiotics, overcoming the issue of antibiotic resistance. In this study, six phages (SAVM01 to SAVM06) were isolated from effluents and were used against a panel of staphylococcal clinical samples isolated from DFUs. A genomic analysis revealed that the phages belonged to the *Herelleviridae* family, with sequences similar to those of the *Kayvirus* genus. No lysogeny-associated genes, known virulence or drug resistance genes were identified in the phage genomes. The phages displayed a strong lytic and antibiofilm activity against DFU clinical isolates, as well as against opportunistic pathogenic coagulase-negative staphylococci. The results presented here suggest that these phages could be effective biocontrol agents against staphylococcal clinical isolates from DFUs.

## 1. Introduction

Diabetes mellitus is a global health problem affecting nearly 10% of the adult population worldwide. Diabetes, associated with vascular pathology and peripheral neuropathy, is responsible for lower-limb complications, such as diabetic foot ulcers (DFUs) [1,2]. The presence of bacterial pathogens in these chronic wounds is one of the main causes of poor healing and spread to bone structures, constituting diabetic foot osteomyelitis (DFOM). The consequences are lower-extremity amputations and a high mortality rate [1,3]. Infected DFUs (DFI) are mainly polymicrobial, with *Staphylococcus aureus* representing the most prevalent bacteria, followed by coagulase-negative staphylococci (CoNS) [4,5].

Whilst the pathogenicity of *S. aureus* is well known, CoNS are often mistakenly considered simple cutaneous commensal bacteria. For instance, *Staphylococcus lugdunensis* is a pathogen responsible for skin and soft tissue infections, sharing more virulence with *S. aureus* than other CoNS [6,7]. However, some CoNS species are now regarded as causal agents of nosocomial infections, such as *Staphylococcus pettenkoferi*, whose virulence and pathogenicity have recently been demonstrated on clinical isolates from DFI, DFOM and the bloodstream [8,9], or *Staphylococcus caprae*, which is responsible for human osteoarticular infections [6,10]. The presence of staphylococci in DFU is therefore highly associated with wound aggravation, including biofilms that delay wound healing [11].

The conventional clinical treatment of DFI requires debridement and antibiotics. Yet these treatments are frequently ineffective because of insufficient vascularization and poor local antibiotic concentrations. Moreover, with the rise of multidrug resistance and the higher tolerance of biofilms towards antibiotics, treatments are becoming more difficult. Therefore, it is becoming necessary to find new therapeutic strategies to treat DFI and enhance the healing process [11,12].

Phage therapy is one alternative strategy developed against bacterial infections. While bacteriophages were discovered over a century ago, their use has only recently regained popularity as a treatment for antibiotic-resistant infections. As lytic phages are viruses of bacteria infecting, multiplying and killing by the act of lysis selectively targeting bacteria at the infection site, the impact on the patient microbiota is minimized. Phages also provide other advantages over antibiotics, such as less significant side effects and a less time-consuming and costly development process [13,14,15]. For therapeutic purposes, strictly virulent phages are preferred in order to prevent lysogeny-related issues. Besides the life cycle, phage genomic information is also required to identify undesirable phage-coded genes, such as integrases, transposases or toxins [16]. For staphylococcal infections, *Kayvirus* are the more represented genus of phages targeting staphylococcal bacteria and represent one of the best control agents. These polyvalent phages display a broad spectrum, infecting mostly *S. aureus* and some CoNS [17,18,19,20]. Interestingly, some have already been used in commercial phage-based preparations to treat DFI [21,22].

In this study, we isolated and characterized new staphylococcal phages for their potential application in DFI treatment. The isolated phages effective against *S. aureus* and CoNS from DFI belong to the genus *Kayvirus*. We also performed morphological, physiological, genomic and antibiofilm characterization.

## 2. Materials and Methods

### 2.1. Bacterial Strains and Growth Conditions

The bacterial strains used in this study belonged to a collection obtained from patients with DFI hospitalized in the Gard-Occitanie Diabetic Foot Clinic (University Hospital of Nîmes, France). Staphylococcal strains including 8 *S. aureus* and 14 CoNS, 3 other Gram-positive species and 2 Gram-negative species were used to determine the phage spectrum. All strains were grown at 37 °C on Tryptic Soy Agar (TSA), in Tryptic Soy Broth (TSB), in Tryptic Soy Broth (TSB) supplemented with 1 mM CaCl_2_ and MgCl_2_ (TSB^+^), in TSA soft overlay supplemented with 1 mM CaCl_2_ and MgCl_2_ or in Brain Heart Infusion (BHI) medium for biofilm assays.

### 2.2. Phage Isolation

Samples (n = 30) collected from several French wastewater treatment plants were used for the isolation of phages. Effluent samples were centrifuged at 5000× *g* for 10 min at room temperature, and the supernatants were filtered through a 0.2 μm filter to remove debris. Then, 3 mL of filtered effluent was mixed with 7 mL of *Staphylococcus* culture in the early logarithmic phase (an optical density at 600 nm (OD_600_ nm) of 0.2). After overnight incubation (180 rpm, at 37 °C), the cultures were centrifuged at 4000× *g* for 10 min, and the resulting supernatants were filtered through a 0.2 µm filter. This enrichment procedure was repeated twice. The presence of phages was verified using the double-layer agar technique. Briefly, 1 mL of enriched sample was mixed with 200 µL of *Staphylococcus* overnight culture and 4 mL of melted soft TSA overlay and poured on a TSA plate. After incubation for 24 h at 37 °C, a single clear plaque was picked up with a Pasteur pipette and added to 4 mL of SM buffer. These steps were repeated three times to perform phage isolation.

### 2.3. Phage Amplification

Phages were amplified using the double-layer agar technique (100 µL of phage suspension, 100 µL of *Staphylococcus* overnight culture, 4 mL of melted soft TSA overlay). The top agar was agitated at 180 rpm for 3 h with 4 mL of SM buffer (100 mM NaCl, 8 mM MgSO_4_, 50 mM Tris HCl (pH 7.5)) to resuspend phage particles. After centrifugation at 4000× *g* for 10 min, the supernatant was filtered twice through a 0.2 µm filter. To define the titer of phage production, 10 µL of decimal dilutions in SM buffer was spotted onto a TSA soft overlay of the bacterial host. The number of phages is expressed as plaque forming units/mL (PFU/mL).

### 2.4. Transmission Electronic Microscopy (TEM) Imaging

Phage particles were sedimented via centrifugation (20,800× *g*, 90 min, 4 °C), and the pellet was further washed in 0.1 M acetate ammonium buffer by repeating the centrifugation step. Subsequently, phage suspensions were dried on a 300-mesh carbon-coated copper grid (Electron Microscopy Sciences, Hatfield, PA, USA) for 2 min, and excess solution was removed using filter paper. The grids were negatively stained with 2% uranyl acetate for 30 s. The grids were air-dried and then imaged via TEM using a JEOL 1400+ microscope operated at 100 kV. Phage sizes were calculated from 20 independent measurements of separated virions using the image processing software Fiji (Version 1.54f), and they are reported as a mean value ± SD.

### 2.5. Phage Whole-Genome Sequencing and Analysis

The phage genomes were sequenced utilizing Illumina paired-end technology using a NovaSeq 6000 S4 (Illumina, San Diego, CA, USA) platform to 100% coverage. Phage whole-genome sequence data are openly available in the NCBI GenBank BioProject PRJNA937056. The phage genomes were assembled de novo using Spades software (version 3.15.4) and blasted against the NCBI GenBank database. For more robustness of phage identification, the fasta sequences were aligned against the Virus database using the Genome Detective Virus Tool platform (https://www.genomedetective.com/, accessed on 12 August 2023). Genome annotation was performed on the DDBJ Fast Annotation and Submission Tool online platform (https://dfast.ddbj.nig.ac.jp/, accessed on 12 August 2023) and illustrated using the Proksee online platform (version 1.0.0) (https://proksee.ca/, accessed on 12 August 2023). Finally, a whole-genome sequence-based phylogenetic tree was constructed using the Orthologous Average Nucleotide Identity Tool (OAT) software (version 0.93.10) [23]. Protein coding sequences, genomic size and GC content were predicted using the RAST^®^ tool [24]. Predicted coding sequences (CDSs) were compared with those in the NCBI BLASTp database, NCBI conserved domain database and HHpred database. A pangenome analysis was performed via a comparison of the annotated phage genomes using Roary tools (version 3.13.0) available on Galaxy online software (https://www.usegalaxy.org.au/, accessed on 12 August 2023) and then visualized on the Phandango online platform [25]. Single-nucleotide polymorphisms (SNPs) based on a whole-genome alignment comparison were identified using the NGPhylogeny online platform (https://ngphylogeny.fr/, accessed on 12 August 2023) and then quantified using snp-dists (version 0.6.3) following an in-house pipeline. An SNP analysis based on sequence position was developed using Snipy (version 4.6.0) available on Galaxy (https://www.usegalaxy.org.au/, accessed on 12 August 2023). Genome representation was performed using the Proksee platform with standard parameters and the CLC Genomics workbench (version 21.0.3) (Qiagen, Redwood City, CA, USA).

### 2.6. Phage Host Range

Phage stocks were serially diluted 10-fold in SM buffer, and 10 μL of phage dilutions (10^8^ and 10^4^ PFU/mL) was spotted onto a TSA soft overlay of the targeted bacteria. The plates were allowed to dry at room temperature before incubation at 37 °C for 24 h. The spot test was considered positive (+) if individual plaques were observed, signifying that bacterial strains were sensitive to the phage. The host range results were verified via a lysis curve assay to confirm the lytic activity of the phages. Briefly, an overnight culture of bacterial isolates was inoculated in TSB^+^, and the culture was incubated at 37 °C 180 rpm until it reached the early exponential growth phase (OD_600_ nm = 0.2). The culture was then infected with phages at various multiplicities of infection (MOI, 10, 1, 0.1 and 0.01) and evaluated using a Tecan apparatus (Tecan, Model Spark, Grödig, Austria GmbH) to monitor the A_600_ nm changes. Data were collected at 20 min intervals for 12 h at 37 °C and 108 rpm.

### 2.7. Biofilm Test

To evaluate the phage control against *Staphylococcus* biofilms, an overnight culture of a *Staphylococcus* strain in BHI broth was first adjusted to an OD_600_ nm of 0.1. Then, 200 µL per well was inoculated in a 96-well polystyrene plate and incubated for 24 h at 37 °C in a humid chamber. The biofilm formed was treated with 200 µL of phage in SM buffer and only buffer for the control. After 24 h of treatment, the biofilm was washed with phosphate-buffered saline (PBS) and air-dried, and crystal violet (0.1%) was added for 20 min at room temperature. To dissolve the biofilm, 100 µL of acid acetic (33%) was added per well for 15 min, and the absorbance was measured with Tecan Apparatus (Tecan, Model Spark, Grödig, Austria GmbH) at A_550_ nm.

### 2.8. Isolation of Phage-Resistant S. aureus Mutants and Sequencing

A culture of *S. aureus* NSA1385 at 10^8^ CFU/mL was mixed with SAVM01 or SAVM02 at 10^8^ PFU/mL (MOI 1) and incubated for 10 min at 37 °C. Subsequently, 4 mL of a melted soft TSA overlay was added to the mixture and poured onto a TSA plate. After 24 h at 37 °C, the mutation rate was estimated by dividing the number of bacterial colonies on each test plate by the initial number of bacteria plated (in triplicate for each phage). Three mutant-resistant colonies generated by SAVM01 and SAVM02 were picked and streaked individually on TSA plates. The resistance was checked by spot test and the phage liquid culturing method with all the SAVM phages as described previously. The genomic DNA of 3 mutants for each phage, as well as the reference strain NSA1385, was extracted and sequenced as previously described to 100% coverage [9], and an in silico analysis was performed according to the “Whole-genome sequencing and analysis” Section.

### 2.9. Statistical Analysis

GraphPad Prism version 6.01 was used to perform a statistical analysis, and the results are indicated in the relevant figure legends.

## 3. Results

### 3.1. Phage Isolation and Morphology

**Figure 1:**
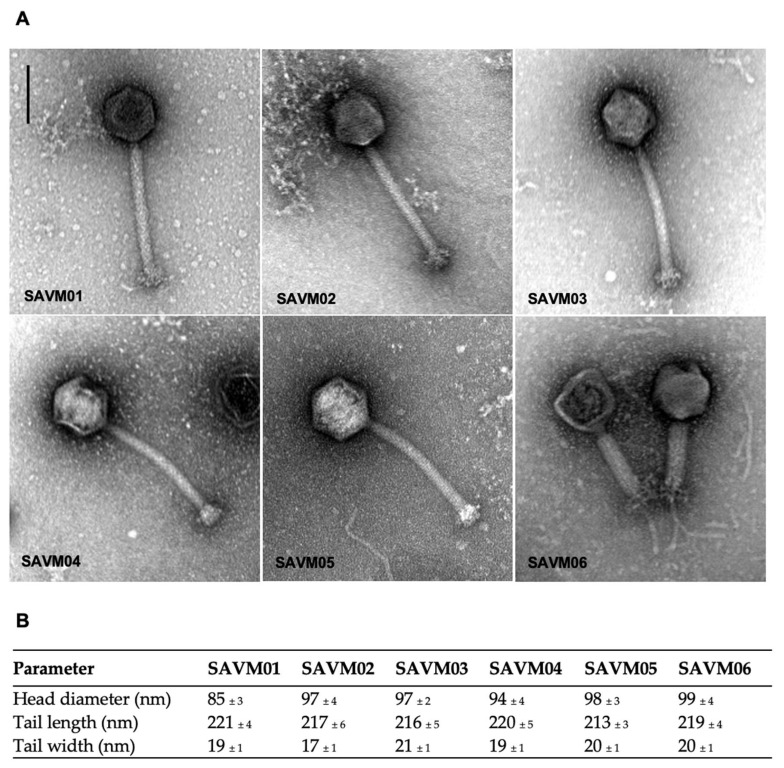
Morphologies of SAVM01 to SAVM06 staphylococcal phages observed using TEM. (**A**) Electron micrographs of phages negatively stained with 1% uranyl acetate show icosahedral capsids with long contractile tails. Scale bar represents 100 nm. (**B**) Measurements (n = 20) of head diameter, tail length and width of virion particles in the extended state were determined with the image processing software Fiji (mean value ± SD).

Effluent samples collected from wastewater treatment plants were used to isolate phages against DFI-associated *Staphylococcus*. Among the several sewage samples, six produced small clear plaques of 1 mm in diameter, which showed the presence of phages and were named SAVM01 to SAVM06. The morphological characteristics of the SAVM phages were assessed using TEM, and it was determined that all phages had similar morphologies, with an icosahedral head ranging from 85 to 99 nm in diameter. The six phages had a long contractile tail, ranging from 213 nm for the smallest one to 221 nm for the largest one. Taken together, the SAVM phages displayed morphological features related to myoviruses. Phage micrographs and phage sizes are summarized in Figure 1.

### 3.2. Phage Whole-Genome Analysis

The full-length phage genome non-redundant sequence assembly generated variable genome sizes from 140,578 bp (SAVM01) to 143,250 bp (SAVM04) and a GC content between 30.2% and 30.4%, respectively. The coding ratio of the total phage genome sequences varied between 89.1 and 91.1%, resulting in a total of 215 protein-encoding genes for SAVM01, 218 for SAVM02, 217 for SAVM03, 220 for SAVM04, and 221 for both SAVM05 and SAVM06 (Figures S1 and S2). Neither rRNA nor CRISPR-specific sequences were identified in the different phage genomes, while four of them (SAVM01-04) contained four genes encoding for tRNA, and only three tRNA genes were identified in the phages SAVM05 and SAVM06 (Supplementary Table S1).

**Figure 2:**
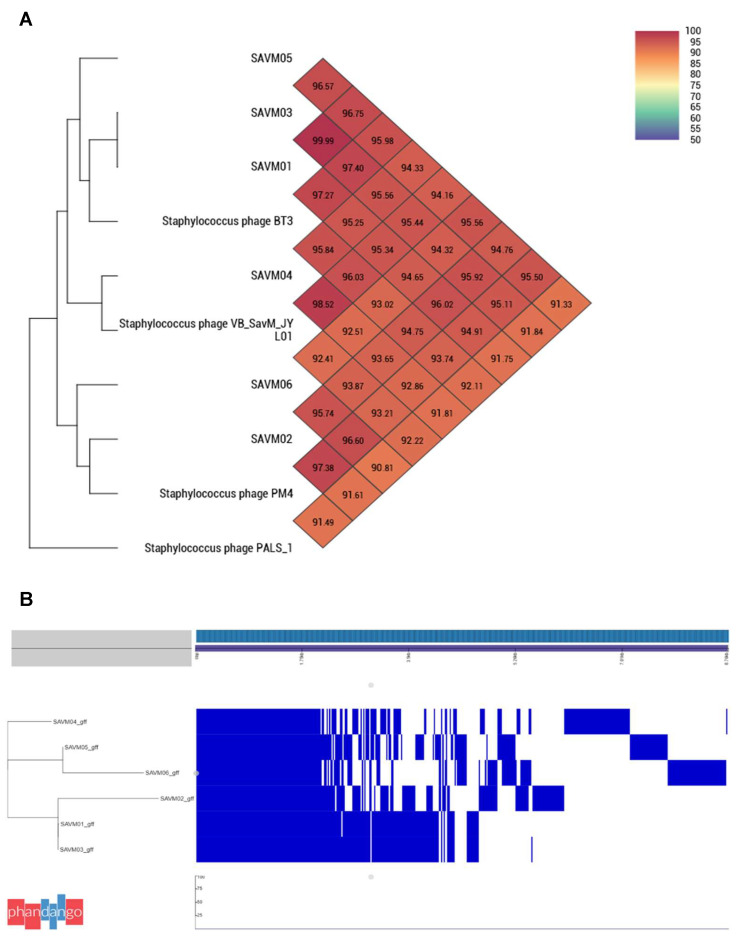
Whole-genome analysis. (**A**) Phylogenetic tree based on whole-genome sequence analysis of the six isolated phages and most representative similar genomes recovered from NCBI GenBank database (accessed on 2 November 2022). Scale represents the percentage of nucleotide identity. (**B**) Pangenome analysis of the six isolated phage genomes. CDS regions of SAVM01 are very similar to those of SAVM03 and present a lower similarity level to those of SAVM02, confirming the phylogenetic analysis results. SAVM04, SAVM05 and SAVM06 display different CDS profiles, explaining the genetic distance observed with other phages.

The phylogenetic analysis based on whole-genome sequences of the isolated phages and other phages collected from the NCBI GenBank database showed that the SAVM phages were related to the *Kayvirus* genus of the *Herelleviridae* family. SAVM01 and SAVM03 showed a 99.99% sequence similarity and shared more than 97% similitude with the *Staphylococcus* phage BT3. The phage SAVM02 demonstrated more similarity to the *Staphylococcus* phage PM4, with a 97.38% sequence similarity, and it shared 96.60% of the genome sequence with SAVM06. SAVM04 shared 98.52% of the genome sequence with the *Staphylococcus* phage VB_SavM_JYL01. The phylogenetic tree was confirmed by the whole-genome annotation and pangenome analysis (Figure 2). The annotated SAVM01 and SAVM03 genomes were highly similar and shared the same encoding genome composition, while a different profile was observed for phages with longer genomes. No SNPs were detected between the SAVM01 and SAVM03 genomes in the SNP distance investigation, whereas more than 6000 SNPs were identified with the other analyzed phages (Supplementary Table S2).

A standard annotation of the pangenomes of the six SAVM phages yielded 105 CDSs shared by all phage genomes. These included the CHAP domain-containing protein known as the anti-*Staphylococcus* protein, killing bacterial cells by cleaving the interpeptide cross-bridge of peptidoglycan [26]. The isolated phages also encoded a metallophosphatase and a Ser/Threonine protein phosphatase involved in energy production and dNTP synthesis with endonuclease activity [27]. The phages encoded for a ribonucleotide diphosphate reductase subunit beta identified as a potential drug target to inhibit *Chlamydia pneumoniae* pathogenicity [28]. Moreover, only the SAVM01, SAVM02 and SAVM06 phages encoded for a lytic transglycosylase slightly homologous to SceD from *S. aureus* known as a secreted virulence factor [29]. Interestingly, this phage protein was previously identified, and it belongs to the kayvirus lytic module and encodes an additional endolysin [30]. Importantly, no bacterial virulence, antibiotic resistance or lysogenic-encoding genes were identified in any of the isolated phages’ genomes.

### 3.3. Phage Host Range and Lytic Activity Analysis

The host range and lytic activity of the SAVM phages were studied using both a spot test and a liquid culturing method. A panel of 34 bacterial strains was used for the tests (Table 1). Interestingly, the SAVM phages were active in approximately the same clinical isolates. Together, the six phages showed lytic activity against *S. aureus* (5/8 isolates), *S. pettenkoferi* (2/4), *S. lugdunensis* (3/4), *S. caprae* (1/4) and *S. haemolyticus* (1/1). However, no lytic effect was observed on the single isolate of *S. epidermidis*. SAVM06 had the narrowest host range, infecting 6 of the 22 staphylococcal isolates, while SAVM01, SAVM03 and SAVM05 were active on 7 isolates, and SAVM04 was active on 8 isolates. Finally, SAVM02 displayed the widest host range, killing half of the staphylococcal isolates. None of the SAVM phages were able to infect other bacterial genera, such as *Enterococcus*, *Streptococcus*, *Corynebacterium*, *Pseudomonas* or *Escherichia*.

**Figure 3:**
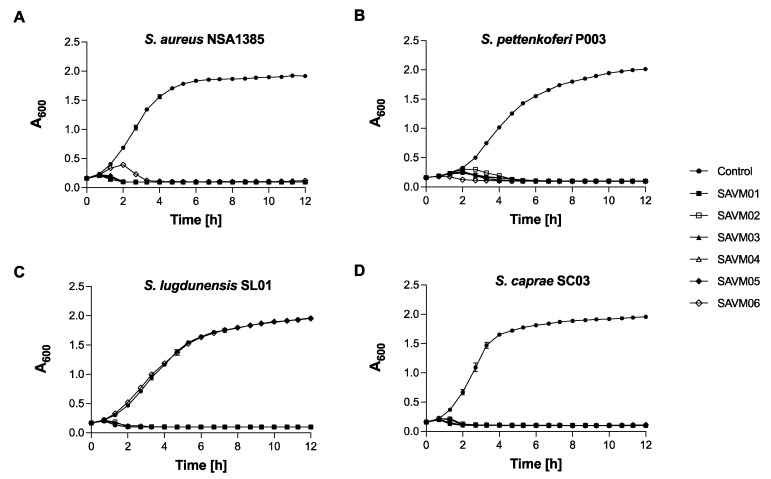
Lytic activity in liquid culture of SAVM01 to SAVM06 against representative *Staphylococcus* strains of DFUs. (**A**) Growth kinetics of *S. aureus* NSA1385, (**B**) *S. pettenkoferi* P003, (**C**) *S. lugdunensis* SL01 and (**D**) *S. caprae* SC03 in TSB^+^ without phage for the control or infected with the 6 phages at MOI 0.1 (10^7^ PFU/mL). Cells were cultured at 37 °C and 108 rpm in 96-well plates using a microplate reader for 12 h. At each time point, the data show the mean A_600_ readings ± SD of three replicates.

To study the phage lytic activity, growth kinetics were performed using staphylococcal strains with or without phage addition. The optimal MOI was first defined on the phage SAVM01 against *S. aureus* NSA1385 using MOIs ranging from 0.01 to 10. As shown in Supplementary Figure S3, the control growth of *S. aureus* NSA1385 without a phage demonstrated a typical growth curve reaching its plateau at 5 h with an absorbance at 600 nm (A_600_nm) of 2.0. When infected by SAVM01 at MOIs 10 and 1, *S. aureus* NSA1385 showed a total growth reduction for 8 h and 10 h, respectively, followed by subpopulation regrowth. The cultures of *S. aureus* NSA1385 infected by SAVM01 at MOIs 0.1 and 0.01 showed an increase in bacterial density during the first 1-2 h, with a subsequent decline in absorbance that remained at 0 for the rest of the experiment. As MOI 0.1 induced a shorter killing latency period than MOI 0.01, and avoided triggering regrowth as in higher MOI, we used MOI 0.1 for our further experiments. Figure 3 presents the growth inhibition triggered by the SAVM phages at MOI 0.1 on four representative species of *Staphylococcus* isolated from diabetic foot samples: *S. aureus* NSA1385, *S. pettenkoferi* P003, *S. lugdunensis* SL01 and *S. caprae* SC03. Each of the SAVM phages exhibited strong lytic activity against the bacterial isolates, represented by a high reduction in absorbance over 12 h compared to the control. The virulence profiles of the phages showed considerable similarities to each other. Like the spot test, SAVM06 showed no inhibitory effect on *S. lugdunensis* SL01 growth in liquid cultures.

### 3.4. Effect of Phage Treatment on Biofilms

**Figure 4:**
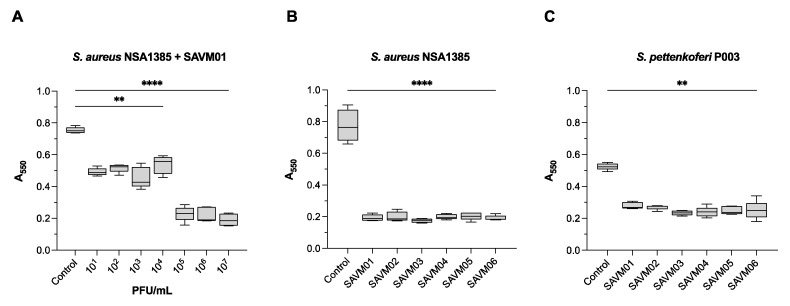
Phage control of a 24 h biofilm quantified using crystal violet staining method (A_550_ nm). (**A**) *S. aureus* NSA1385 biofilm was treated by SAVM01 at various concentrations (10^1^-10^7^ PFU/mL) and incubated for another 24 h at 37 °C to determine the optimal phage concentration. (**B**) *S. aureus* NSA1385 and (**C**) *S. pettenkoferi* P003 biofilms were treated with SAVM01-SAVM06 phages at 10^7^ PFU/mL and incubated for another 24 h at 37 °C. For the control, biofilms were treated with SM buffer. Each box plot represents the mean ± SD of five replicates. **, *p* < 0.01; ****, *p* < 0.0001 (ordinary one-way ANOVA test).

In order to determine the antibiofilm capacity of the isolated phages, we performed biofilm tests using the crystal violet staining method. SAVM01 was first tested to evaluate the optimal phage concentration able to disrupt a 24 h preformed biofilm of *S. aureus* NSA1385 (Figure 4). Compared to the biofilm control treated with an SM buffer, SAVM01-treated biofilms were decreased by ~35% at 10^1^ to 10^4^ PFU/mL and ~73% at 10^5^ to 10^7^ PFU/mL. Thus, the optimal phage concentration required to significantly reduce a preformed biofilm was 10^7^ PFU/mL, which was then used in further experiments. The next tests were performed on two DFI isolates, *S. aureus* NSA1385 and *S. pettenkoferi* P003, using the six SAVM phages at 10^7^ PFU/mL (Figure 4). After phage treatment, biofilms were successfully disrupted by 75% for *S. aureus* NSA1385 and 52% for S. *pettenkoferi* P003, indicating an important biofilm reduction. Interestingly, the antibiofilm activity was very similar for each of the SAVM phages.

### 3.5. S. aureus Phage-Resistant Mutant Analysis

In order to investigate bacterial resistance to the SAVM phages, we decided to isolate and analyze phage-resistant mutants of *S. aureus* NSA1385 (the strain sensitive to the six SAVM phages). Mutants were first generated using SAVM01 to verify whether they truly displayed resistance after streak purification. We confirmed that the bacterial mutants generated from SAVM01 were still resistant after picking and streaking on agar plates, suggesting that their growth was triggered by stable or heritable phage resistance rather than a transient phenotype. A cross-resistance spot test subsequently demonstrated that the SAVM01-resistant mutants were also resistant to the other SAVM phages, except for SAVM02. This finding implies that SAVM02 probably targets different resistance mechanisms than the five other SAVM phages. In addition, the same mutant generation steps were repeated with SAVM02 and *S. aureus* NSA1385. The first noticeable difference was the lower rate of resistant colonies for SAVM02 than SAVM01, namely, 1.6 × 10^−7^ and 4.3 × 10^−6^, respectively, after 24 h of plate incubation. Interestingly, after streaking the SAVM02-resistant colonies, the cross-resistance spot test showed that these mutants exhibited resistance to SAVM02 and to all other SAVM phages. It is noteworthy that the resistant colonies had similar morphologies to the wild-type colonies of *S. aureus* NSA1385 (i.e., beige, round and about 2 mm in size) and displayed small colony variants (SCVs) enclosed in the soft agar overlay.

The SAVM01 and SAVM02 phage-resistant mutants were sequenced in order to identify the mutations related to phage resistance, and the sequences were aligned with those of the reference genome of the parental strain *S. aureus* NSA1385. We decided to focus only on the mutations common to all three sequenced mutants. Therefore, for the SAVM01-resistant mutants, a total of 14 mutations were identified, while SAVM02-resistant mutants had 42 common mutations (including insertions, SNPs and complex substitutions) (Supplementary Table S3). Interestingly, the 14 mutations detected in the SAVM01-resistant mutants were found at the same positions in the SAVM02 mutants. Most of the mutations induced by the two phages were in the *lexA* gene (10/14; 23/42). Among these, seven resulted in amino acid substitutions in the protein sequence, while the others were found to be silent mutations. The additional mutations shared between the SAVM01- and SAVM02-resistant mutants had an impact on the glucosaminidase domain-containing protein with four amino acid substitutions, as well as an insertion in the *recJ* gene resulting in amino acid changes and a stop codon shift causing protein extension from 35 to 758 amino acids. The specific mutations in the SAVM02-resistant mutants included the *essG*, *xerC* and *splA* genes. In addition, the DnaD domain-containing protein and the RusA crossover junction endodeoxyribonuclease were absent in the three SAVM01-resistant mutants compared with the parental strain (Supplementary Table S3).

## 4. Discussion

DFIs are a serious complication of diabetes mellitus causing lower-limb amputations and increasing morbidity and mortality. *S. aureus* and CoNS are the predominant bacteria isolated from these chronic wounds. These bacteria are characterized by their ability to form biofilms, as well as by the high prevalence of multidrug-resistant strains. These aspects greatly complicate the treatment of DFI [1,11]. Phages are considered a potential alternative strategy to antibiotics. However, studies on phage therapy for staphylococcal infections often focus on *S. aureus*. Only a few studies have investigated the isolation and the biological and genomic characterization of phages infecting the clinical isolates of CoNS, especially *S. epidermidis* [17,31,32,33]. In this study, we isolated and characterized six phages (SAVM01 to SAVM06) from wastewater treatment plants using a selection of *Staphylococcus* spp. clinical strains from DFI. Despite being isolated from various effluent sources, our genomic analysis revealed that the six SAVM phages were closely related to each other, notably SAVM01 and SAVM03, which showed a 99.99% sequence similarity. TEM imaging also revealed that the phages were morphologically similar and exhibited features related to myoviruses [34]. According to the phage taxonomy update, the SAVM phages belonged to the *Kayvirus* genus of the *Herelleviridae* family [35]. In terms of potential medical applications, phages associated with these taxa are regarded as the most interesting phages. Indeed, kayviruses have already demonstrated their efficacy in the treatment of various *S. aureus* infections, both in animal models and human clinical cases (see phages VB-SavM-JYL01 [36], and K, Sb-1, vB_SauH_2002 and cocktail AB-SA01 [14]). In addition, the absence of lysogeny-associated genes in their genomes is consistent with the International Committee on Taxonomy of Viruses, which reports that phages belonging to the *Herelleviridae* family are strictly lytic [37]. This criterion is essential to avoid horizontal gene transfer phenomena responsible for the spread of resistance/virulence markers. The SAVM phages also lacked known virulence or antibiotic resistance genes; if they did not, the phages could not be considered for therapeutic application [38].

Once it was confirmed that the newly isolated phages were strictly virulent and safe to be applied as phage treatments, we determined their activity on clinical staphylococcal isolates from DFI. While several methods are used for host range and lytic activity determination, a spot test and the phage liquid culturing method are the most commonly used [39]. The use of both techniques is complementary and allows for some drawbacks to be circumvented. A false negative result may occur in the case of poor phage diffusion in a double agar overlay, as well as in the cases of abortive infections, “lysis from without” or endolysins [40,41,42]. Using these tests, we determined that the SAVM phages were specific to *Staphylococcus* spp., including *S. aureus* and opportunistic pathogenic CoNS from DFI. It was found that SAVM02 exhibited the broadest host range, infecting almost half of the staphylococcal strains tested. Regarding other studies, *Kayvirus* are described as polyvalent phages displaying important lytic activity against *S. aureus* mainly. In particular, recently isolated and characterized kayviruses demonstrated an ability to infect almost all clinical strains of multidrug-resistant *S. aureus* and showed only minor activity on CoNS [19,20]. The authors described bacterial inhibition at high phage concentrations on some CoNS but without plaque formation at decreasing titers (i.e., lysis from external events without phage infection) [19]. Taking this into account, the SAVM phages seemed to display stronger activity within the different staphylococcal species. Furthermore, the SAVM phages displayed important lytic activity controlling the bacterial growth of different *Staphylococcus* spp. over 12 h in the liquid culturing method. It is noteworthy that the optimal MOI was a key parameter to allow for rapid and constant bacterial inhibition. While phages at higher MOI produce an earlier reduction in bacterial growth, they result in a selection pressure generating bacterial regrowth through the acquisition of temporal immunity or the growth of a resistant subpopulation [39].

Moreover, the clinical impact of biofilms represents a challenge for DFU management. Indeed, biofilms are involved in 60 to 80% of DFUs with a high risk of lower-limb amputations in diabetic patients. One of the main causes of treatment failure is the high tolerance of biofilms towards antibiotics [11]. While antibiotics cannot diffuse through the polymeric matrix of a biofilm, it has already been shown that kayviruses can effectively penetrate and disrupt *S. aureus* and *S. epidermidis* biofilm structures [43,44,45,46]. We showed that the SAVM phages reduced biofilm formation on representative DFU isolates, namely, *S. aureus* NSA1385 and *S. pettenkoferi* P003. The similar antibiofilm activities between the six phages demonstrates once again their close relationship. Taken together, our findings are consistent with those of previous research indicating that phage treatment for 24 h with myoviruses allows for a significant reduction in *S. aureus* biofilms [47].

To understand the bacterial evolution under phage selection pressures, phage-resistant mutants of *S. aureus* NSA1385 were fully sequenced and analyzed in a comparative genomic study. We showed that SAVM01 and SAVM02 both induce a high number of mutations in the *lexA* gene, responsible for the repression of a number of genes involved in the response to DNA damage (SOS response) [48]. Once DNA damage has been induced by stress, the RecA protein interacts with single-stranded DNA to promote LexA cleavage via autoproteolysis, thus activating SOS gene expression. Two sites completely conserved in the LexA superfamily are involved in self-cleavage [49], but none of the seven mutations that caused amino acid substitutions in the LexA protein sequence impacted these sites. Moreover, the SAVM01 and SAVM02 phage-resistant mutants were also found to have a mutation in the *recJ* gene, which is related to DNA damage repair and the encoding of exonucleases that degrade single-stranded DNA. The insertion of a single nucleotide base led to the shift of the stop codon, extending the protein from 35 to 758 amino acids and leading to its possible inactivation.

Concerning mutations specific to SAVM02-resistant mutants, we observed four amino acid substitutions in the glucosaminidase domain-containing protein, which plays a role in the hydrolysis of peptidoglycan glycosidic bonds. Chan et al. showed that mutations in the N-acetylglucosaminidase genes of *S. aureus* Newman had no impact on wall teichoic acid (WTA) synthesis but did have an impact on replication, causing a slightly reduced rate. The mutation of the glucosaminidase B gene (*sagB*) may also lead to a small increase in vancomycin resistance in *S. aureus* USA300 [50]. However, phage resistance induced by mutations in this gene has never been studied, and it remains unclear whether the substitution of the two amino acids changes the protein function. The SAVM02-resistant mutants were also mutated in the *xerC* gene, which is related to DNA damage repair and the encoding of a site-specific recombinase XerC; the *essG* gene related to a component of the type VII secretion system (Ess); and the *splA* gene encoding a serine protease. However, phage resistance mechanisms have never been explored in these genes. Finally, we found two missing genes in the SAVM01-resistant mutants compared with the parental strain and SAVM02-resistant mutants. The first codes the DnaD protein involved in primosome function for the recruitment of the replication fork helicase onto the DNA, and the second codes the RusA endonuclease involved in genetic recombination and DNA repair. Nevertheless, no study has shown that phage resistance can be linked to the deletion of these genes.

Taken together, the six studied *Kayvirus* SAVM01 to SAVM06 phages represent promising candidates for therapeutic purposes due to their wide host range.

## Figures and Tables

**Table 1:** Phage host range determined using spot test of SAVM01 to SAVM06 on 32 clinical strains isolated from infected DFUs and on 2 laboratory strains (*).

Species	Isolates	Antibiotic Resistance	SAVM					
			01	02	03	04	05	06
***Staphylococcus* strains**								
*Staphylococcus aureus*	USA300 JE2 *	ERY, OXA	-	+	-	-	-	-
	SH1000 *	WT	-	+	-	-	-	-
	NSA1385	TET	+	+	+	+	+	+
	SARM141	PEN, OXA, LIN, PRI, OFX	-	-	-	-	-	-
	SASM148	OFX	-	-	-	-	-	-
	SAC1	PEN, ERY	-	+	-	-	-	-
	SAI3	PEN, OXA, GEN, ERY, VAN	-	-	-	-	-	-
	SAC4	PEN, ERY	-	-	-	+	-	+
*Staphylococcus pettenkoferi*	SP165	PEN, OXA, ERY, CMN, LIN, OFX, RIF, FOS	+	+	+	+	+	+
	P003	WT	+	+	+	+	+	+
	P009	PEN, ERY, CMN, LIN, PRI, SYN, TET, OFX, FUS, FOS	-	-	-	-	-	-
	P023	PEN	-	-	-	-	-	-
*Staphylococcus lugdunensis*	SL137	PEN	-	+	-	-	-	-
	Nim.SL.01	WT	+	+	+	+	+	-
	Nim.SL.02	WT	-	-	-	-	-	-
	Nim.SL.03	FOS	+	+	+	+	+	-
*Staphylococcus caprae*	SC108	PEN, FUS, FOS	-	-	-	-	-	-
	Nim.SC.01	FOS	-	-	-	-	-	-
	Nim.SC.02	ERY, FOS	-	-	-	-	-	-
	Nim.SC.03	FOS	+	+	+	+	+	+
*Staphylococcus haemolyticus*	SH82	FOS, FUS	+	+	+	+	+	+
*Staphylococcus epidermidis*	SE163	PEN, OXA, ERY, TET, OFX	-	-	-	-	-	-
**Gram-positive bacteria**								
*Enterococcus faecalis*	Nim.EF.01	CMN, SXT	-	-	-	-	-	-
	Nim.EF.02	CMN, SXT	-	-	-	-	-	-
*Streptococcus agalactiae*	Nim.StA.01	TET	-	-	-	-	-	-
*Corynebacterium striatum*	Nim.CS.04	PEN, GEN, CMN, SXT, CIP	-	-	-	-	-	-
**Gram-negative bacteria**								
*Pseudomonas aeruginosa*	PAC1	ATM	-	-	-	-	-	-
	PAC2	PIP, ATM, CAZ	-	-	-	-	-	-
	PAC4	WT	-	-	-	-	-	-
*Escherichia coli*	Nim.EC.01	WT	-	-	-	-	-	-
	103	AMX, AMC, TIC, SXT	-	-	-	-	-	-
	104	AMX, AMC, TIC, FOX	-	-	-	-	-	-

AMX, amoxicillin; AMC, amoxicillin/clavulanate; ATM, aztreonam; CAZ, ceftazidime; CIP, ciprofloxacin; CMN, clindamycin; ERY, erythromycin; FOS, fosfomycin; FUS, fusidic acid; FOX, cefoxitin; GEN, gentamicin; LIN, lincomycin; OFX, ofloxacin; OXA, oxacillin; PEN, penicillin G; PIP, piperacillin; PRI, pristinamycin; RIF, rifampicin; SXT, Co-trimoxazole; SYN, synergystin; TET, tetracycline; TIC, ticarcillin; VAN, vancomycin; WT, wild type. According to EUCAST recommendations (https://www.eucast.org/clinical_breakpoints/, accessed on 12 August 2023). (-) not phage-sensitive; (grey +) phage-sensitive.

## Data Availability

On reasonable request, the corresponding author will provide the datasets produced and analyzed in the present work.

## Supplementary Materials

The following supporting information can be downloaded at https://www.mdpi.com/article/10.3390/v15122287/s1, Table S1: Genome analysis of phages SAVM01 to SAVM06; Table S2: Single-nucleotide polymorphism (SNP) distance based on whole-genome analysis; Figure S1: Phage genome representation; Figure S2: Pairwise genome alignment and phage genome identification; Figure S3: Lytic activity of the phage SAVM01 at various MOIs; Table S3: Identification of bacterial mutations using whole-genome sequencing analysis of phage-resistant S. aureus NSA1385 mutants.
