# Supplementary material for: Isolation and Characterization of New Bacteriophages against Staphylococcal Clinical Isolates from Diabetic Foot Ulcers
Source: Viruses. 2023 Nov 22;15(12):2287. doi: 10.3390/v15122287 (PMC10747802; doi:10.3390/v15122287)

Table S1. Genome analysis of the phages SAVM01 to SAVM06.

Genome statistics	SAVM01	SAVM02	SAVM03	SAVM04	SAVM05	SAVM06
Total Length (bp)	140,578	141,431	140,717	143,250	142,363	141,167
No. of Sequences	1	1	1	1	1	1
GC Content (%)	30.4	30.4	30.4	30.2	30.4	30.4
N50	140,578	141,431	140,717	143,250	142,363	141,167
Gap Ratio (%)	0.0	0.0	0.0	0.0	0.0	0.0
No. of CDSs	215	218	217	220	221	221
No. of rRNA	0	0	0	0	0	0
No. of tRNA	4	4	4	4	3	3
No. of CRISPRS	0	0	0	0	0	0
Coding Ratio (%)	89.1	89.9	90.3	91.1	90.1	90.0

Table S2. Single nucleotide polymorphism (SNP) distance based on whole genome analysis.

SNP distance	SAVM01	SAVM02	SAVM03	SAVM04	SAVM05	SAVM06
**SAVM01**	0	6370	0	8616	6361	10,248
**SAVM02**	6,370	0	6370	11,008	9174	7777
**SAVM03**	0	6370	0	8616	6361	10248
**SAVM04**	8616	11,008	8616	0	9766	12499
**SAVM05**	6361	9174	6361	9766	0	8720
**SAVM06**	10,248	7777	10248	12499	8720	0

Table S3. Identification of bacterial mutations by whole-genome sequencing analysis of phage-resistant *S. aureus* NSA1385 mutants.

Phage to which mutant are resistant	Position	Reference	Mutation	Annotation	Protein alteration
SAVM01	1649353	A	AT	*recJ* gene Single-stranded-DNA-specific exonuclease RecJ	Tyrosine → Isoleucine Codon stop shift → protein extension (35 Aa → 758 Aa)
	1928613	A	G	Glucosaminidase domain-containing protein	
	1928619	AGCATT	TGTATG		Alanine → Threonine Asparagine → Histidine
	2065350	A	G	Putative HNHc nuclease	
	2071806	T	C	*lexA*_4 gene LexA repressor	
	2071836	ATAT	GTAC		
	2071845	C	T		
	2071851	GCAT	ACAC		
	2071869	T	C		
	2071878	CGAAA	TGAAG		Threonine → Alanine
	2071905	T	C		
	2071912	G	A		Valine → Isoleucine
	2071917	TGGT	CGGC		
	2071944	A	G		
SAVM02	292849	T	C	*essG*_4 gene TIGR01741 family protein	
	292858	C	T		
	292867	C	A		Serine → Arginine
	292900	A	T		
	1494182	C	T	DUF1381 domain-containing protein	
	1507177	A	T	*xerC*_2 gene Site-specific recombinase XerC	Isoleucine → Phenylalanine
	1507194	CAAAAATAAC	TAAGAACAAT		
	1507209	TAAATAC	GAAGTAT		
	1649353	A	AT	*recJ* gene Single-stranded-DNA-specific exonuclease RecJ	Tyrosine → Isoleucine Codon stop shift → protein extension (35 Aa → 758 Aa)
	1847183	T	TGTTAAAATA	*splA* gene Serine protease splA	Addition of 3 amino acids: Isoleucine, Lysine, Threonine
	1926867	C	A	BppU family phage baseplate upper protein	
	1928613	A	G	Glucosaminidase domain-containing protein	
	1928619	AGCATT	TGTATG		Alanine → Threonine Asparagine → Histidine
	1928643	TCTTGC	CCTAGA		Alanine → Serine

1928656	T	C		Lysine → Arginine
1954480	GC	AT	DUF3269 family protein	
1959882	T	C	DUF1108 family protein	Lysine → Arginine
2065350	A	G	Putative HNHc nuclease	
2071676	TT	CA	lexA_4 gene LexA repressor	Phenylalanine → Serine
2071683	A	G		
2071689	A	G		
2071695	CCGCCAAGAAAAC	AAGACAAAGCAAT		Glutamic Acid → Serine
2071713	T	C		
2071719	TGCAAATGAACAATTGGAA	CGCGAATAGTCAGTTAGAT		Glutamic Acid → Serine<br>Glutamic Acid → Aspartic Acid
2071743	G	A		
2071766	G	A		Glycine → Glutamic Acid
2071776	G	A		
2071782	AC	GT		
2071788	A	G		
2071800	A	G		
2071806	T	C		
2071821	CATA	TATC		
2071836	ATAT	GTAC		
2071845	C	T		
2071851	GCAT	ACAC		
2071869	T	C		
2071878	CGAAA	TGAAG		Threonine → Alanine
2071905	T	C		
2071912	G	A		Valine → Isoleucine
2071917	TGGT	CGGC		
2071944	A	G		
2570775	T	C	Intergenic region	

Figure S1. **(A)** Unique phage genome representation using Phaster online software available on http://phaster.ca/ with standard parameters. The phage genome size is > 140kb and composed on 217 proteins including 215 phage hit proteins with a majority of phage hypothetical proteins, with 30,53% CG %. Phage like proteins, fiber proteins, attachment site, and coat proteins are the most identified proteins by annotation. **(B)** Phage genome representation using Proksee (version 1.0). The phage SAVM05 genome was used as reference and compared with the other five genomes. The annotation of the phage genomes was performed using Pharokka and added directly on genome plots.

(A)

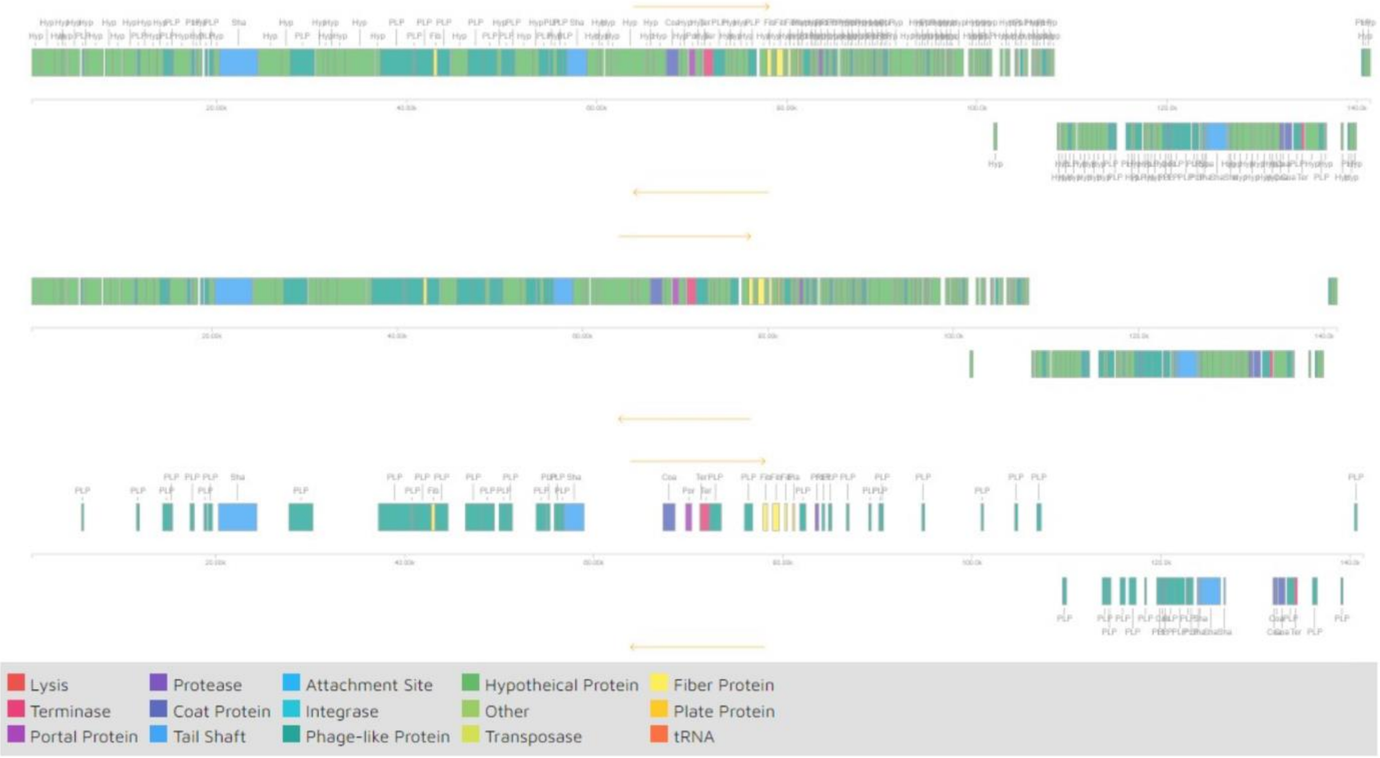

(B)

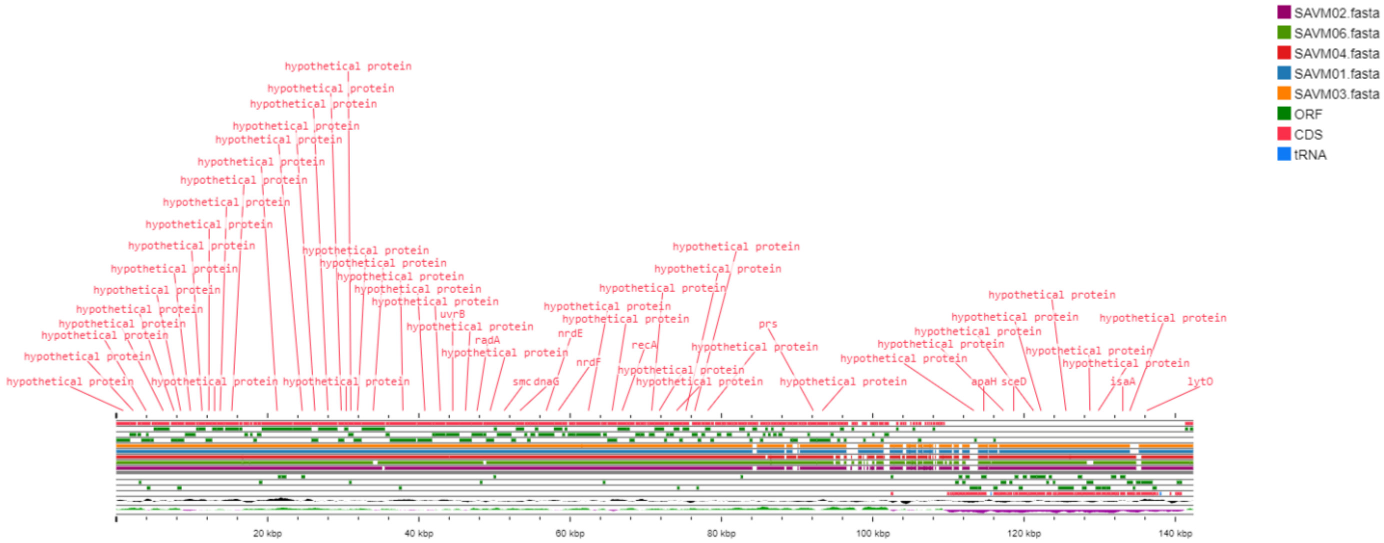

Figure S2: Pairwise genome alignment and phage genome identification. **(A)** Heatmap representing the pairwise genome alignment. **(B)** Phage genome identification using genome detective plateform (Genome Detective.com).

(A)

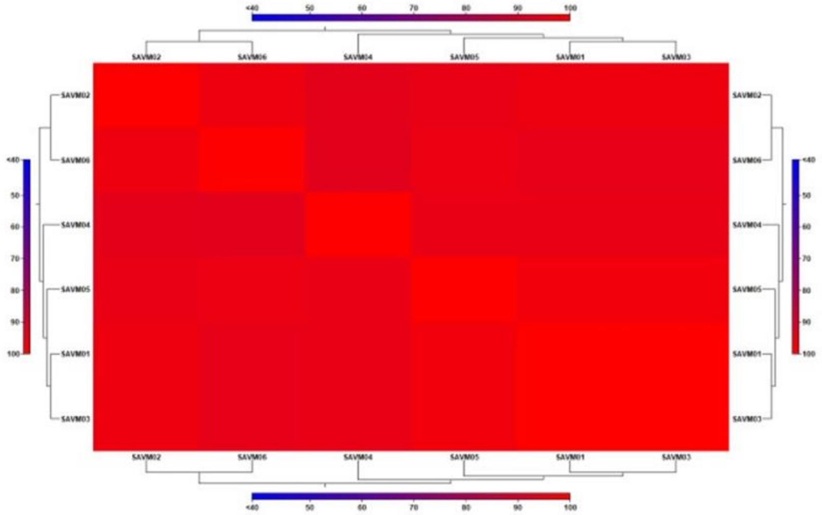

(B)

Name	Length	Report	Species	NT / AA Identity (%)		Genome
SAVM01	140578	Report	Kayvirus rodi	95.9622	96.8291	
SAVM02	141431	Report	Staphylococcus phage K	96.0514	96.6092	
SAVM03	140717	Report	Staphylococcus phage Staph1N	95.1594	96.3217	
SAVM04	143250	Report	Staphylococcus phage G15	96.2907	97.8493	
SAVM05	142363	Report	Kayvirus rodi	94.5375	96.4286	
SAVM06	141167	Report	Staphylococcus phage 812	96.8384	97.5296	

Figure S3. Lytic activity of the phage SAVM01 at various MOI. *S. aureus* NSA1385 was infected by SAVM01 phage at MOI of 10, 1, 0.1 and 0.01 (10^6-10^9 PFU/mL) or treated by SM buffer for the control, and cultured for 12 h at 37 °C and 108 rpm in 96-well plates using a microplate reader. At each time point, the data show the mean A_{600} readings ± SD of three replicates.

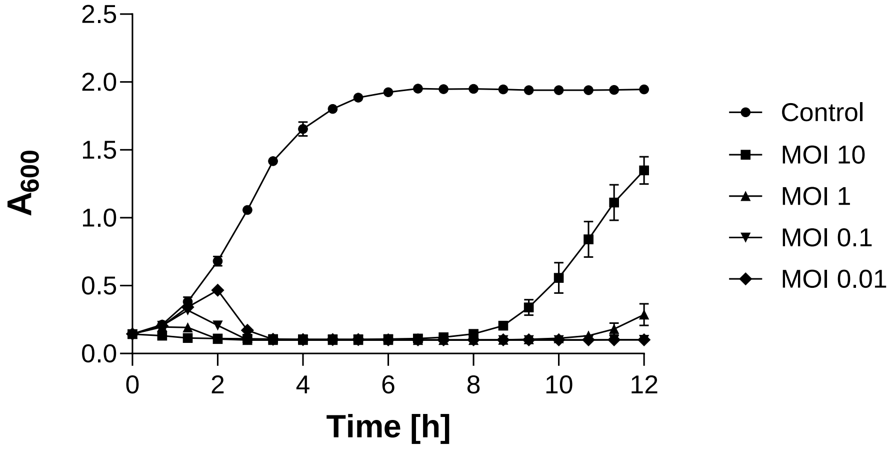

Supplement: Supplementary file 1 [file viruses-15-02287-s001.zip › viruses-2674393-supplementary.pdf]
